# Finding unusual peptides on the internet using plain three letter sequence codes

**DOI:** 10.1186/1758-2946-5-S1-P49

**Published:** 2013-03-22

**Authors:** Alexander Kos, Hans-Jürgen Himmler

**Affiliations:** 1 AKos GmbH, Steinen, D-79585;Germany

## 

Finding peptides with modified amino acids is difficult or impossible when you use plain three letter sequence codes and BLAST. You can find those peptides when you use the structure as a query, but drawing the structure correctly is rather difficult for non-chemists. We developed CWM Global Search [1] with Proteax [2]. This is an Internet search engine that allows scientists such as biologists to input plain three letter sequence codes and subsequently search the corresponding chemical structures on the Internet including substructure searches and structure similarity searches.

The results are mapped back to three letter sequence codes if possible. This makes the interpretation of the search results much easier than trying to interpret the structures or systematic names normally provided in the databases. Results can also be easily compared using "Proteax for Spreadsheets".

We demonstrate how to input the plain three letter sequence codes in the Proteax editor and the easy and powerful interpretation of the results performing substructure and structure similarity searches for peptides in PubChem and ChEBI.

**Figure 1:**
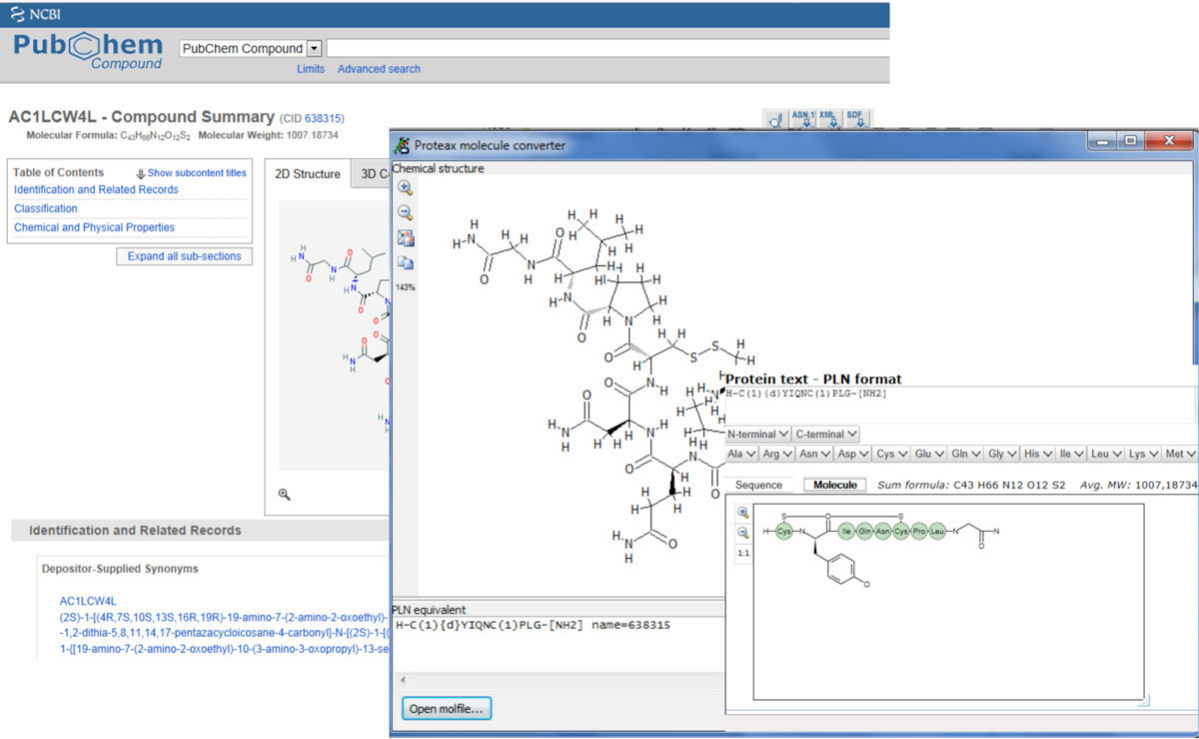
**Result from a substructure search is converted in an easy to read three letter sequence code**. Unsystematic amino acids are shown as structures.
